# The Role of Histone H4 Biotinylation in the Structure of Nucleosomes

**DOI:** 10.1371/journal.pone.0016299

**Published:** 2011-01-27

**Authors:** Nina A. Filenko, Carol Kolar, John T. West, S. Abbie Smith, Yousef I. Hassan, Gloria E. O. Borgstahl, Janos Zempleni, Yuri L. Lyubchenko

**Affiliations:** 1 Department of Pharmaceutical Sciences, University of Nebraska Medical Center, Omaha, Nebraska, United States of America; 2 The Eppley Institute for Research in Cancer and Allied Diseases, University of Nebraska Medical Center, Omaha, Nebraska, United States of America; 3 Department of Nutrition and Health Sciences, University of Nebraska, Lincoln, Nebraska, United States of America; 4 The University of Oklahoma Health Sciences Center, Oklahoma City, Oklahoma, United States of America; University of South Florida College of Medicine, United States of America

## Abstract

**Background:**

Post-translational modifications of histones play important roles in regulating nucleosome structure and gene transcription. It has been shown that biotinylation of histone H4 at lysine-12 in histone H4 (K12Bio-H4) is associated with repression of a number of genes. We hypothesized that biotinylation modifies the physical structure of nucleosomes, and that biotin-induced conformational changes contribute to gene silencing associated with histone biotinylation.

**Methodology/Principal Findings:**

To test this hypothesis we used atomic force microscopy to directly analyze structures of nucleosomes formed with biotin-modified and non-modified H4. The analysis of the AFM images revealed a 13% increase in the length of DNA wrapped around the histone core in nucleosomes with biotinylated H4. This statistically significant (p<0.001) difference between native and biotinylated nucleosomes corresponds to adding approximately 20 bp to the classical 147 bp length of nucleosomal DNA.

**Conclusions/Significance:**

The increase in nucleosomal DNA length is predicted to stabilize the association of DNA with histones and therefore to prevent nucleosomes from unwrapping. This provides a mechanistic explanation for the gene silencing associated with K12Bio-H4. The proposed single-molecule AFM approach will be instrumental for studying the effects of various epigenetic modifications of nucleosomes, in addition to biotinylation.

## Introduction

Modifications of histones are among the epigenetic marks that influence gene expression. Distinct histone modifications of one or more tails have been proposed to act sequentially or in combination to form a ‘histone code’ that is read by other proteins to bring about distinct downstream events [1]. Posttranslational modifications of histone tails include methylation [2], phosphorylation [3], acetylation [4], ubiquitination [5] and biotinylation [6]. Our understanding of the molecular and structural mechanisms of how these modifications impact transcriptional activity remains inadequate. The demonstration that acetylation of histones affects chromatin compaction at the mononucleosomal [7] and trinucleosomal [8] levels provided initial mechanistic insight into the relationship between nucleosome structure and gene expression. Using fluorescence resonance energy transfer (FRET) analysis, Gansen et al. demonstrated that histone acetylation decreased stability of mononucleosomes [9]. Histone H4 acetylation at lysine 16 (K16Ac-H4) was shown to impact chromatin structure by inhibiting the formation of compact 30-nanometer–like fibers and to impede the ability to form cross-fiber interactions [10]. In addition, K16Ac-H4 inhibits the chromatin assembly process and interferes with the function of the ATP-dependent chromatin assembly and remodeling factor, ACF. Recently, single-pair FRET was used to probe conformational changes in mononucleosomes induced by DNA methylation [11]. These studies showed that CpG methylation leads to the compaction of nucleosomes and nucleosome structural rigidity.

Most recently, a novel posttranslational modification of histones, biotinylation, was discovered by one of the co-authors [6], [12], [13] and independently confirmed in another laboratory [14]. More recently, using LC/MS/MS, a third laboratory detected large quantities of biotinylated histone H4 in *Candida albicans* [15]. Initially, a mechanism for enzymatic catalysis of histone biotinylation by biotinidase was proposed by Wolf and co-workers based on *in vitro* studies [16]. However, recent studies used recombinant histones and holocarboxylase synthetase (HCS) to unambiguously demonstrate that HCS has histone biotinyl ligase activity [17], and it is now evident that biotinylation of histones is mediated preferentially by HCS [18]. Biotinylated histones have been detected in human cells [13] and distinct histone biotinylation sites were defined using peptide and *in vivo* studies [6], [12]. Ten distinct histone biotinylation sites have been identified: five in histone H2A, three in histone H3 and two in histone H4. Histone H4 can be biotinylated at amino terminal lysines 8 (K8Bio-H4) and 12 (K12Bio-H4) [6].

Several lines of evidence suggest a functional role for histone biotinylation in gene silencing, cellular responses to DNA damage, and cell proliferation as reviewed elsewhere [19]. Briefly, K8bio-H4 and K12bio-H4 localize to alpha-satellite repeats in pericentromeric regions, as well as to transcriptionally repressed chromatin loci [20]. K12bio-H4 is highly enriched in telomeric repeats from human lung IMR-90 fibroblasts, where one out of three H4-histones is biotinylated at K12 [21]. Low abundance of biotinylation marks has been linked with cleft palate in mice [14] and genome instability in humans [22].

Based on the biochemical evidence above, we hypothesized that H4 biotinylation alters the structure of nucleosomes and reduces the accessibility of DNA to transcriptional machinery. Biophysically testing this concept was a major goal of this paper. We have recently shown that high-resolution AFM imaging can detect the subtle conformational changes in nucleosomes and reveal their dynamic character [23], [24]. In the current work, the same AFM technology was employed to quantify histone biotinylation-dependent changes in nucleosome structure. We report that K12-biotinylation in histone H4 causes a significant change in nucleosome structure leading to a ∼15% increase in the amount DNA wrapped around nucleosomes. We propose that this effect provides a partial mechanistic explanation for the correlation between histone biotinylation and gene silencing.

## Results

### Experimental design

**Figure 1:**
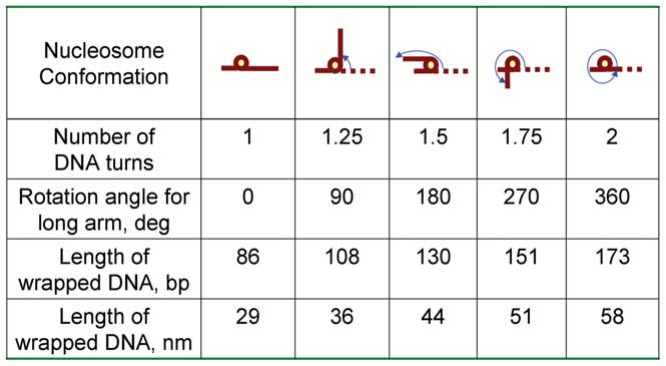
Schematics for various stages of the nucleosome unwrapping. Nucleosome conformations are shown with different number of turns, rotation angle and length of wrapped DNA.

Similar to previous studies [23], [24], the DNA template designed for this work was a fragment of 353 bp DNA containing the 147 bp nucleosome positioning 601 sequence [25], flanked by two arms of different lengths (79 bp and 127 bp). Differential arm lengths enables mapping of the nucleosome position [25]. Depending on the number of DNA turns around the histone core, the nucleosome will adopt one of several different morphologies shown schematically in Fig. 1.The initial design corresponds to the complex with one turn and the four other conformations correspond to complexes with 1.25, 1.5, 1.75 and 2 turns. For clarity, Fig. 1 shows rotation of the long arm only, although uniform wrapping of both arms occurs starting at a position in the center of the 147 bp region, so the length of the arms gradually decreases upon DNA wrapping. In addition, DNA wrapping is accompanied by changes in the interarm angle. We assigned a rotation angle of zero to the position of the long arm for the complex with one turn. The conformation with 1.25 DNA turns is characterized by a 90° rotation angle, and the complexes with 1.5, 1.75 and 2 turns have the rotation angles 180°, 270° and 360°, respectively. These parameters were used in the procedure of assigning of the nucleosome core particle (NCP) conformation.

Previous studies have demonstrated that histone proteins produced in *E. coli* are competent to form nucleosomes with DNA *in vitro.* For AFM studies we required significant quantities of purified H4 with and without the biotin mark at K12. In addition, we needed to be able to verify that biotinylation of H4 was only present at the twelfth residue and not at alternate or additional sites. Previous studies suggested that the *E. coli* HCS ortholog BirA has histone biotinyl ligase activity and that recombinant histones produced in bacteria could be biotinylated [26]. Since the N-terminal tail of histone H4 is solvent-exposed and contains several lysine residues [27] that could be biotinylated by BirA, we used site-directed mutagenesis to convert the codon for K12 to that for Cys. Importantly, this mutation introduces the only Cys in the entire recombinant H4 sequence. After expression in *E. coli*, the undesired BirA-biotinylated minor fraction of recombinant histone could then be removed from lysates by avidin chromatography, and the unbiotinylated major fraction was subjected to sulfhydryl-specific biotinylation of cysteine-12 in K12C-H4 in a chemical reaction with maleimide-PEG_2_-biotin (K12Cbio-H4). The level of chemical biotinylation of K12C-H4 was assessed by Western blotting with streptavidin conjugates (Fig. S1, panel a) and anti-biotin antibodies (Fig. S1, panel b). A faint biotin signal is detectable in K12C-H4 purified from *E. coli* prior to avidin chromatography (Fig. S1b, lane 1), consistent with low-level *E. coli B*irA biotinylation of the heterologous H4 protein. No band was observed in the purified fraction of histone K12C-H4, whereas a strong signal was produced by chemical biotinylation with maleimide-PEG_2_-biotin (Fig. S1, lane 3, both panels a and b). Protein identity was confirmed using anti-H4 (Fig. S1, panel c). Nucleosomes were formed with the 601 positioning sequence (described above) in the presence of biotinylated K12CBio-H4 or alternately with K12C-H4 that was not subjected to the maleimide reaction. The other histone components, H2A, H2B, and H3 were derived from *E. coli* and purchased from NEB. Wild-type or K12-H4 was also acquired from NEB and was used in nucleosome preparations to control for structural changes induced by the Cys substitution.

### AFM imaging of mononucleosomes

**Figure 2:**
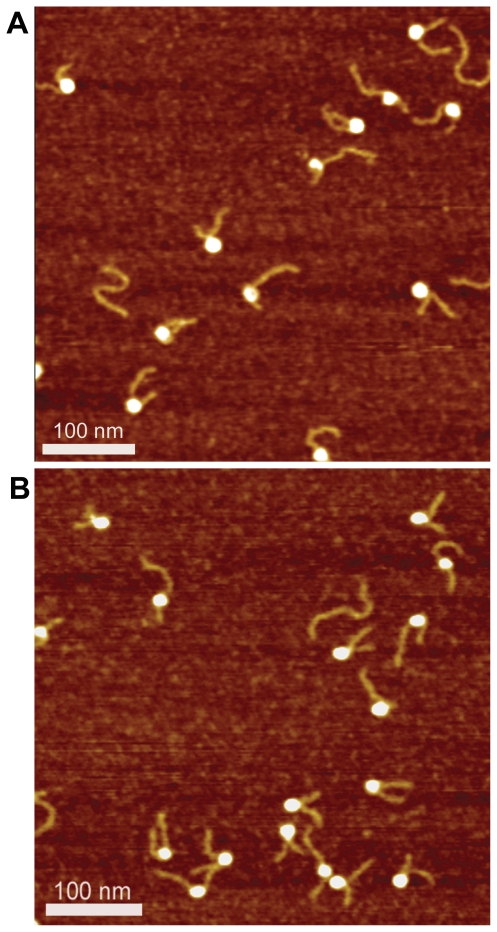
Representative AFM scans of nucleosome core particles. Nucleosomes were reconstituted using native histone H4 (a) or biotinylated histone K12Cbio-H4 (b). Images were acquired with NanoScope IIId AFM system operating in Tapping mode. Scan sizes are 0.5 µm.

**Figure 3:**
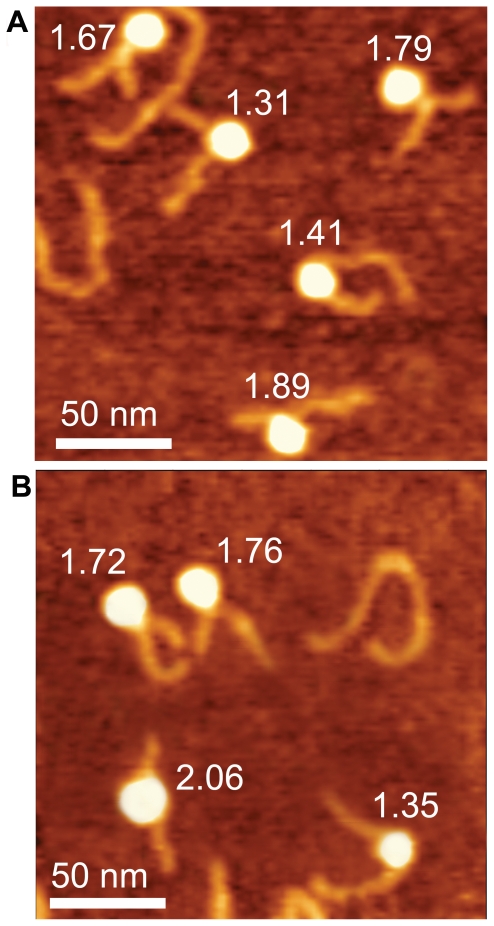
Representative enlarged AFM scans of NCP. Nucleosomes were reconstituted using non-biotinylated native histone H4 (a) and biotinylated K12C histone H4 (b). The complexes are labeled with the number of DNA turns around histone octamers. Scans sizes are 200×200 nm.

As previously [24], nucleosomes were deposited on APS-mica, rinsed, dried and imaged with AFM in air (Fig. 2 and Fig. S2). In AFM images, the nucleosomes are visible as bright globules with the DNA arms extending from both sides of the particles. Samples prepared with WT histones and those with the use of K12C H4 were very similar. The yield of nucleosome sample in these samples was 70–80% with the rest being the naked DNA. The morphology of NCP is different when assembled from biotin-free, native histone H4 as compared to those with biotinylated histone, K12Cbio-H4. For instance, the number of molecules with 1.7–1.75 turns with crossed DNA arms is 17% (2 molecules out of 12 total) and 31% (5 molecules out of 16 total), for the nucleosome samples with native H4 and K12Cbio-H4, respectively. This is further illustrated in Fig. 3 where enlarged images for the native and biotinylated nucleosomal samples are shown. These images can be interpreted in terms of different number of DNA turns around the histone core, the number of DNA turns are marked next to the nucleosome particles. The analysis of the images as described below enabled us to characterize the structure of nucleosomes in a number of the nucleosomal DNA turns (see Materials and Methods S1, section II for details). The molecule with 1.31 turn has a 110°rotation angle, (110°+360°)/360°  = 1.31. The molecule with 1.41 turn has almost parallel arms and rotation angle of 147°. The arms of the molecules with 1.79 and 1.76 turns are crossed with a rotation angle of 283° and 275°, respectively. The arms form almost a straight line with rotation angle of 380° in particle with 2.06 turns of DNA. Therefore, biotinylated nucleosomes (Fig. 3b) compared to native nucleosome population (Fig. 3a) is enriched with complexes with a large number of turns with the mean value of turns being 1.61 and 1.72 for native and biotinylated nucleosomes, respectively.

### Analysis of AFM data

To assess the effect of biotin on the structure of nucleosome we measured the following parameters of nucleosomes over a large number of AFM images: (1) the length of the two protruding DNA arms and (2) the angle between the DNA arms [23], [24]. Then the number of DNA turns around the histone core octamer was calculated, The length of nucleosomal DNA wrapped around octamer (nsDNA) was calculated by subtracting the lengths of both free DNA arms from total length of uncomplexed DNA (see Fig. S3).

**Figure 4:**
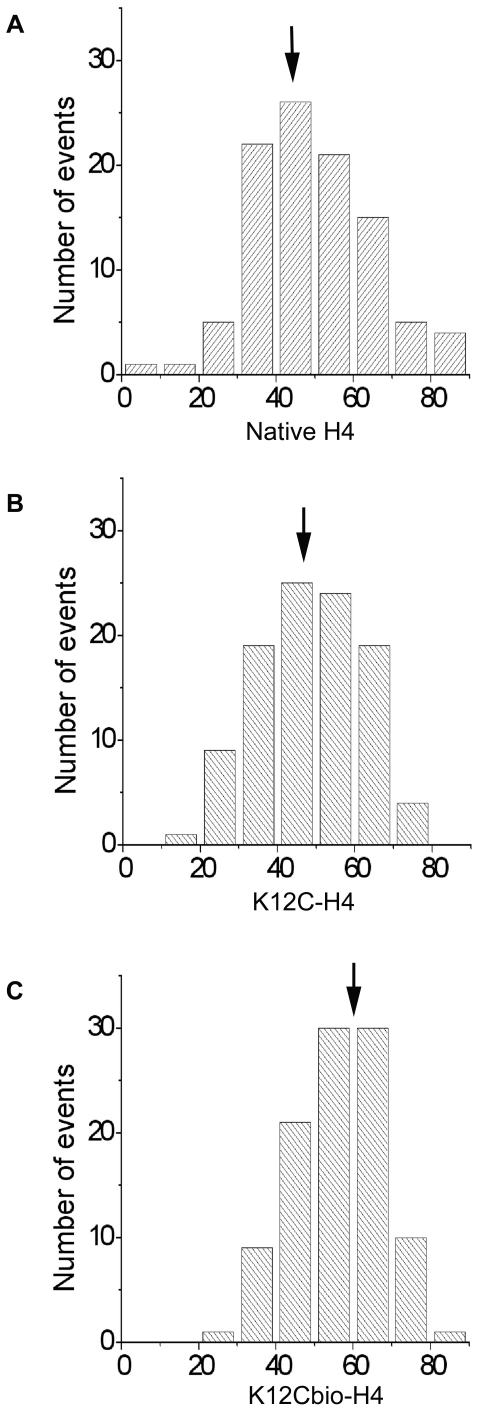
Histograms for lengths of nucleosomal DNA (nsDNA) wrapped around histone cores. Nucleosomes were reconstituted using native H4 histone (a), K12C-H4 mutant (b) or K12Cbio-H4 histone (c). It can be seen that in nucleosomes made with K12Cbio-H4 wDNA is shifted towards higher value compared to samples reconstituted using non-biotinylated native H4 or K12C-H4 mutant. The mean values of nsDNA indicated with arrows were 49.8 nm ±1.5 nm, 48.8 nm ±1.4 nm and 56.6 nm ±1.1 nm for NCP containing native histone H4, K12C-H4 mutant, and K12Cbio-H4, respectively.

Figure 4 compares the distribution of nsDNA length in K12Cbio-H4 nucleosomes to those in native H4 and K12C-H4 controls. Each dataset was in the range of 100–110 complexes. While lengths of nsDNA were similar in nucleosomes with native H4 (panel a) and K12C-H4 (panel b), 49.8±1.5 nm, 48.8±1.4 nm respectively, the length of nsDNA was greater in sample K12Cbio-H4 (panel c), 56.6±1.1 nm. The difference between samples native H4 and K12Cbio-H4 equaled 6.8±2.6 nm, which is larger then the sum of standard errors and is statistically significant (p<0.001), with degree of freedom of 198). The length difference of nsDNA is ∼20 bp in the length and is equivalent to extra 0.2 turns of nsDNA or to the increase of the number of turns per octamer from 1.75 to almost 2 turns.

The effect of the histone H4 biotinylation on nucleosome structure was reproducible (Fig. S4). Mean values for the nsDNA lengths for native nucleosomes and K12Cbio-H4 NCP were 47.5±1.3 and 54.2±1.2 nm, respectively for second independent set of samples. In this set of measurements the average difference in the length of nsDNA was 6.7±2.5 nm, which is statistically identical to the 6.8±2.6 nm obtained for the first set.

We confirmed our findings by using an alternative approach in which the value of angle between the arms is used to calculate the number of DNA turns [23], [24]. Table 1 summarizes the results based on angle measurements for two independent experiments. The number of DNA turns in nucleosomes was greater for K12Cbio-H4 compared with H4 and K12C-H4 controls. The proportion of molecules with more than 1.5 turns was 55, 56 and 73% in samples H4, K12C-H4 and K12Cbio-H4, respectively. Table 2 shows the differences in number of nucleosomal turns for DNA between the native and biotinylated nucleosomes calculated with both methods. The difference is 0.2 turns of nsDNA for both sets of native and biotinylated nucleosomes when calculated based on angle measurements. Thus, both procedures reproducibly yielded similar results: biotinylation increases the length of DNA associated directly with the nucleosome by ∼13% that leads to the increase in the mean number of DNA turns in nucleosomes from 1.75 (native) to about 2 (biotinylated mutant).

**Table 1:** Comparison of number of turns of nucleosomal DNA (nsDNA) obtained from two independent samples of native H4 and biotinylated H4 (K12Cbio-H4) nucleosomes.

	Native H4 ncp, #1	Native H4 ncp, #2	K12Cbio-H4, sample #1	K12Cbio-H4, sample #2
**Number of turns of nsDNA**	1.62±0.05	1.61±0.04	1.82±0.04	1.81±0.06

**Table 2:** Differences in the number of nucleosomal turns for DNA between nucleosomes assembled using native H4 and biotinylated K12Cbio-H4.

The number of turns of nsDNA	K12Cbio-H4 - native H4, sample set #1	K12Cbio-H4 - native H4, sample set #2
**Based on the length of nsDNA**	0.23±0.09	0.23±0.08
**Based on the angle measurements**	0.20±0.09	0.20±0.10

## Discussion

**Figure 5:**
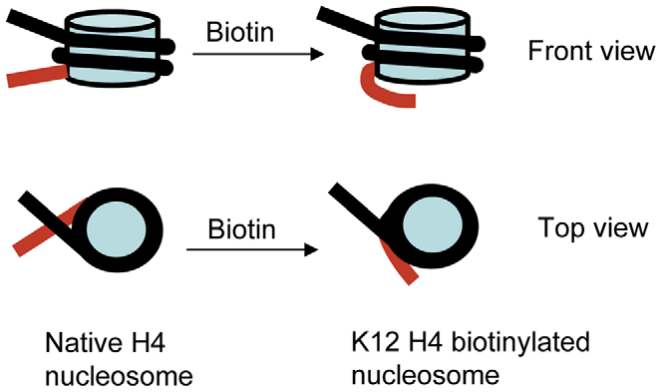
Model of the effect of biotinylation on conformation of nucleosome. Both front and top views are shown. The segment of the DNA arm that contributes to additional wrapping is shown in red.

This work shows directly and unambiguously that biotinylation of histone H4 at K12 leads to a statistically significant increase in the length of DNA wrapped around the histone core octamer. This change of the nucleosome structure is shown schematically in Fig. 5. Compared to 147 bp length of nsDNA wrapped around nucleosomes formed with non-biotinylated wt H4 or with K12C-H4, biotinylation at position 12 increases the length of nsDNA to an average of 167 bp, which corresponds to adding to nsDNA of almost 0.2 nucleosomal turns. Such a substantial increase of the length of wrapped DNA should lead to elevated stability of nucleosomes and is congruent with functional studies demonstrating a role for histone biotinylation in transcriptional repression. The conclusion on elevated stability of nucleosomes with increased number of turns is supported by our recent time-lapse AFM imaging data [23], [24] on the dynamics of nucleosomes. These data showed that nucleosomes with 2 turns are much more stable than those with 1.7 turns. Therefore, biotinylation of H4 at position 12 leads to stabilization of nucleosomes, suggesting that this structural change contributes to regulation of gene expression.

Similar to previous studies [23], [24], individual nucleosomes containing control H4 and K12Cbio-H4 vary in the number of DNA turns around each histone core suggesting that biotinylated nucleosomes, similar to controls, dynamically undergo transient unwrapping-wrapping processes. However, comparison of the histograms from all samples (Fig. 4) reveals that biotinylation causes a uniform shift towards more condensed nucleosomal structures across the entire histogram without a preferable shift to any particular conformation. This observation suggests that biotinylation of H4 does not lead to the formation of nucleosomes with a particular number of turns, but rather that biotin-containing nucleosomes maintain the ability to undergo condensation and decondensation with a more condensed average structure.

Based on crystallography data, the well-ordered domains in histones mediate the strong interactions of the histone core with DNA, but the N-terminus of histone H4 is unstructured and does not contribute to DNA binding. We propose that biotinylation stabilizes the structure of the N-terminus of histone H4, leading to the formation of novel contacts with DNA and the other histones that accommodate two additional DNA pitches in the nucleosomal body. The magnitude of this effect is surprisingly high, given that only one residue (C12) in one histone protein (H4) was biotinylated. Indeed, biotin is capable of forming of stable complexes with proteins and complexes of biotin with avidin and streptavidin are among the strongest noncovalent molecular associations (K_d_∼10^−15^ M). According to crystallographic data for biotin-avidin complexes, an array of polar and aromatic residues in avidin is involved in the tight binding [28]. Several aromatic residues such as tryptophan, phenylalanine and tyrosine, in the biotin-binding site of avidin form a “hydrophobic box”, in which the biotin molecule resides. As histones also possess aromatic and polar amino acids, similar attractive interactions can be formed between biotin and histone molecules within the nucleosome particle. There are a large number of potential candidates for such interactions, and crystallography studies are needed to test this model. Apparently the significant change in nucleosome structure can increases nucleosome stability and thus provides additional contacts for binding of DNA leading to increasing of the stability of nucleosomes or alternately provides novel binding sites for repressive epigenetic factors. Therefore we speculate that the elevated stability of nucleosomes due to the increase of the length of nsDNA is at least partially responsible for silencing of genes reported in previous biological activity studies [20],[21],[29].

We used a K12C mutant of histone H4 in our studies for targeted biotinylation of position 12, while in vivo only lysine residues are biotinylated. Our AFM studies suggest that nucleosomes composed of native H4 and non-biotinylated K12C-H4 had similar conformations, implying that the K12C substitution does not alter the conformation of H4. Thus it is likely that changes in NCP conformation are solely due to the biotinylation mark. Note that chemical biotinylation scheme used in this work (via PEG linker) is different from the in vivo biotinylation in which biotin is bound to the epsilon amino group of lysine. The difference in the linker may contribute to the structural change of the nucleosome, but the finding that biotin is required for the observed effect suggests that biotinylation *per se* rather than the chemical bond is critical in the nucleosome structural change.

In conclusion, we should add that studies during the past decade have dramatically changed our view of the structure of chromatin and of its key unit, the nucleosome, in particular. A static picture is currently being replaced with a dynamic one, and single-molecule techniques were instrumental in characterizing these dynamic properties of nucleosomes. AFM is capable of characterizing complex molecular system at the nanoscale level making it possible to visualize directly the unwrapping process of nucleosomes. The current work highlights the ability of AFM to identify structural changes in nucleosomes induced by a local modification, such as biotinylation, and thus paves the way for studies of effects of other epigenetic modifications of nucleosomes.

## Materials and Methods

### Preparation of mutant histone H4

Amino acid lysine at position 12 (K12) in histone H4 was mutated to cysteine (K12C-H4), using Quick-change mutagenesis (Stratagene) according to manufacturer's instructions, to generate a target for subsequent chemical biotinylation with a sulfhydryl-reactive reagent. The coding sequence of H4 histone from *Xenopus laevis* in a pET3a vector system was used as a template. The primers were [29] 5′-GGTAAAGGTGGTAAA GGTCTGGGTTGCGGTGGTGCTAAACGTCAC-3′ and (antisense) 5′-GTGACGTTTAGCACCGCAACCCAGACCTTTACCACCTTTACC-3′ (corresponding to protein sequence KGGKGLGCGGAKRH). pET3a-transformed *E. coli* strain BL21(DE3) (Novagen) was grown to abs_600_  = 0.8 in 2XYT medium, and the expression of K12C-H4 was induced with 0.4 mM IPTG at 37°C for 90 min. The cell pellet was lysed by Emulsiflex in wash buffer (50 mM Tris HCl, pH 7.5; 100 mM NaCl, 1 mM 2-mercapthoethanol) and centrifuged at 23,000 g for 10 min at 4°C. The inclusion body pellet was washed in wash buffer containing 1% Triton X-100. The pellet was suspended in 1 ml dimethyl sulfoxide, stirred 30 min at RT and wash buffer containing 6 M guanidine hydrochloride was added. K12C-H4 was purified on Superdex200 HiLoad 16/60 column, Prep Grade (GE Healthcare).

### Biotin-depletion of H4 histone

Previous studies suggested that microbial *Bir*A has enzymatic activity to biotinylate recombinant histones, albeit at low levels [26]. Endogenously biotinylated K12C-H4 was removed using avidin agarose resin (Pierce). Briefly, 3 mg of K12C-H4 in PBS buffer were added to 2 ml of 50% resin slurry in PBS (equivalent to 1 ml of settled gel) and incubated overnight at 4°C with shaking. The sample was centrifuged for 1 min at 5000× g and the supernatant, containing biotin-depleted histone, was used for subsequent studies in amount of 1 mg at concentration0.3 mg/ml.

### Chemical biotinylation of K12C-H4

K12C-H4 was biotinylated at C12 residue to produce K12Cbio-H4 by using the sulfhydryl-reactive reagent Maleimide-PEG2-Biotin according to the manufacturer's instructions (Thermo Scientific). Note that histone H4 contains no cysteine residues other than the C12 inserted by mutation. Before biotinylation, any C12-C12 disulfide bonds between two K12C-H4 molecules were reduced with 5 mM *tris*(2-carboxyethyl)phosphine (TCEP) for 30 min at RT. After TCEP removal with Microcon centrifugal filters (Millipore), molecular weight cutoff 3,000, a 20-fold molar excess of Maleimide-PEG2-Biotin was added and samples were incubated at 4°C overnight. The protein was purified from nonreacted Maleimide-PEG2-Biotin using Microcon filters with molecular weight cutoff 3,000.

### Preparation of nucleosomal DNA

DNA for nucleosome assembly was generated by PCR using plasmid pGEM3Z-601 as a template, which codes for a high-affinity nucleosome positioning sequence [30]. The PCR reaction (33 cycles of 94°C/30 s, 54°C/30 s, 72°C/30 s) was conducted in buffer containing 2.5 mM MgCl_2_, 0.15 mM dNTPs and 0.016U/µl of Taq DNA polymerase with the following primers: forward primer 5′-GEMf CGGCCAGTGAATTGTAATACG-3′; reverse primer GEMr 5′-CGGGATCCTAATGACCAAGG-3′.

### Histone octamer assembly and purification

Histone octamers were assembled as follows [31]. Procedure of histone octamer preparation is given in supplementary materials. Octamers were separated from tetramer and dimer fractions with size-exclusion chromatography (SEC) with Superdex 200 PC 3.2/30 column (GE Healthcare) at 4°C. SEC fractions were analyzed for purity and histone stoichiometry using SDS-PAGE. The gel was stained using Coomassie Blue stain. Fractions containing histones H2A, H2B, H3 and H4 in approximately equal ratios were pooled and concentrated by centrifugation at 10,000 g. See specifics in Materials and Methods S1, section I.

### Nucleosome refolding

Nucleosomes were prepared as described [31]. Briefly, histone octamers and DNA containing the nucleosome positioning sequence were mixed in equimolar concentrations in 2 M NaCl and kept for 30 min at RT. A dilution series was prepared using 10 mM Tris HCl to produce final concentrations of 1 M, 0.67 M, and 0.5 M NaCl. Diluted samples were kept at 4°C for 1 h before dialysis against one change of volume of 0.2 M NaCl overnight. Nucleosomes were concentrated using Microcon centrifugal filter device, MWCO 10,000 at 7,000 g for 10 min at 4°C and dialyzed against one change of 200 ml of buffer containing 10 mM Hepes-NaCl, pH 7.5, and 1 mM EDTA for 3 h at 4°C.

### Atomic force microscopy

Freshly cleaved mica was modified with 167 µM solution of 1-(3-aminopropyl)-silatrane (APS) for 30 min at room T to make APS-mica as described previously in [32]. Other AFM works on the chromatin in addition to APS functionalization used mica coated with poly-lysine [8] or spermidine [33]. The nucleosome stock solution was diluted into 10 mM Tris-HCl, pH 7.5, 4 mM, MgCl_2_ buffer and 5 µl of the solution were deposited on APS-treated mica for 3 minutes, washed with deionized water and dried under argon flow. AFM images were collected on NanoScope IIId system (Veeco/Digital Instruments, Santa Barbara, CA) as described in [23] and [24].

### Measurement of nucleosome parameters

The samples deposited on APS mica were analyzed with Femtoscan software. The following 5 initial parameters were measured: length of each DNA arm, angle between arms (interarm angle), height of nucleosome core particle and diameter as width of nucleosome core particle at half height. The length of DNA was measured with FemtoScan software using parameter “curve”. The length of wrapped DNA was measured by subtracting sum of both DNA arms from length of uncomplexed DNA. Importantly, the analysis of one set of native and biotinylated nucleosomes was performed blindly without disclosing whether the nucleosome contained biotinylated on non-biotinylated H4. The errors of the calculated mean values are standard errors of the mean (SEM).

### Assumptions for estimation of the number of DNA turns

The calculations of DNA turns wrapped around histone octamers were based on the following assumptions. (1) Based on crystallographic data, 147 bp of DNA are wound around histone octamer in 1.7 turns [34], i.e., 1 turn contains 86 bp of DNA. (2) As long as in B form of DNA one base pair corresponds to 0.34 nm, the expected length value for 1 turn is 29 nm. How specific number of turns was assigned is explained in detail in Materials and Methods S1.

## Supporting Information

Figure S1 **Testing of different samples of K12C H4 histone for biotinylation state.** K12C-H4 was purified after overexpression in *E.coli* (lane1), depleted for fraction possibly biotinylated in vivo at lysines (lane 2) and biotinylated *in vitro* at cysteine 12 with Maleimide-PEG2-Biotin (lane 3). The level of chemical biotinylation was assessed by Western blotting with streptavidin conjugates (panel a) and anti-biotin antibodies (panel b). Control western blot with anti-H4 antibodies (panel c) demonstrates that all three samples in lanes 1-3 are histone H4. M - marker.
(TIF)

Figure S2 **Representative AFM scan of nucleosome core particles reconstituted with K12C H4 mutant.** Nucleosomes were made with K12C-H4 histone mutant. The sample was prepared and imaged as described for Figure 2. The image represents nucleosomes with different amount of DNA wrapped around the core particle. K12Cbio-H4 nucleosome conformation is similar to nucleosomes made with native histone H4. Scan size is 0.5 µm.
(TIF)

Figure S3 **Length of uncomplexed DNA.** Shown is distribution of length of uncomplexed DNA used for reconstitution of nucleosome core particles. The length of DNA was measured with FemtoScan software using parameter “curve”. The data were plotted as statistical histogram and fitted with Gaussian distribution. The most probable value of 117.5±0.1 nm was taken as length of full DNA molecule in subsequent calculations of length of DNA wrapped around nucleosome.
(TIF)

Figure S4 **Comparison of wrapped DNA length from two independent samples of native and biotinylated H4 nucleosomes.** Nucleosomes were reconstituted using native H4 histone (a) or biotinylated K12Cbio-H4 histone (b). Length of free DNA was measured using Femtoscan software. The length of wrapped DNA was calculated by subtracting the sum of both free DNA hands from total length of DNA. Data from two independent experiments are overlapped. It can be seen that in nucleosomes made with K12Cbio-H4 wDNA is shifted towards higher value compared to samples reconstituted using non-biotinylated native H4. Mean values for native NCP wDNA were 49.8±1.5 and 47.5±1.3 nm, respectively. Mean values for wDNA of biotinylated NCP (K12Cbio-H4) were 56.6±1.1 and 54.2±1.2 nm, respectively.
(TIF)

Materials and Methods S1 Supplement to Materials and Methods.
(DOC)
