# Supplementary figures and images for: The Role of Histone H4 Biotinylation in the Structure of Nucleosomes
Source: PLoS One. 2011 Jan 27;6(1):e16299. doi: 10.1371/journal.pone.0016299 (PMC3029316; doi:10.1371/journal.pone.0016299)

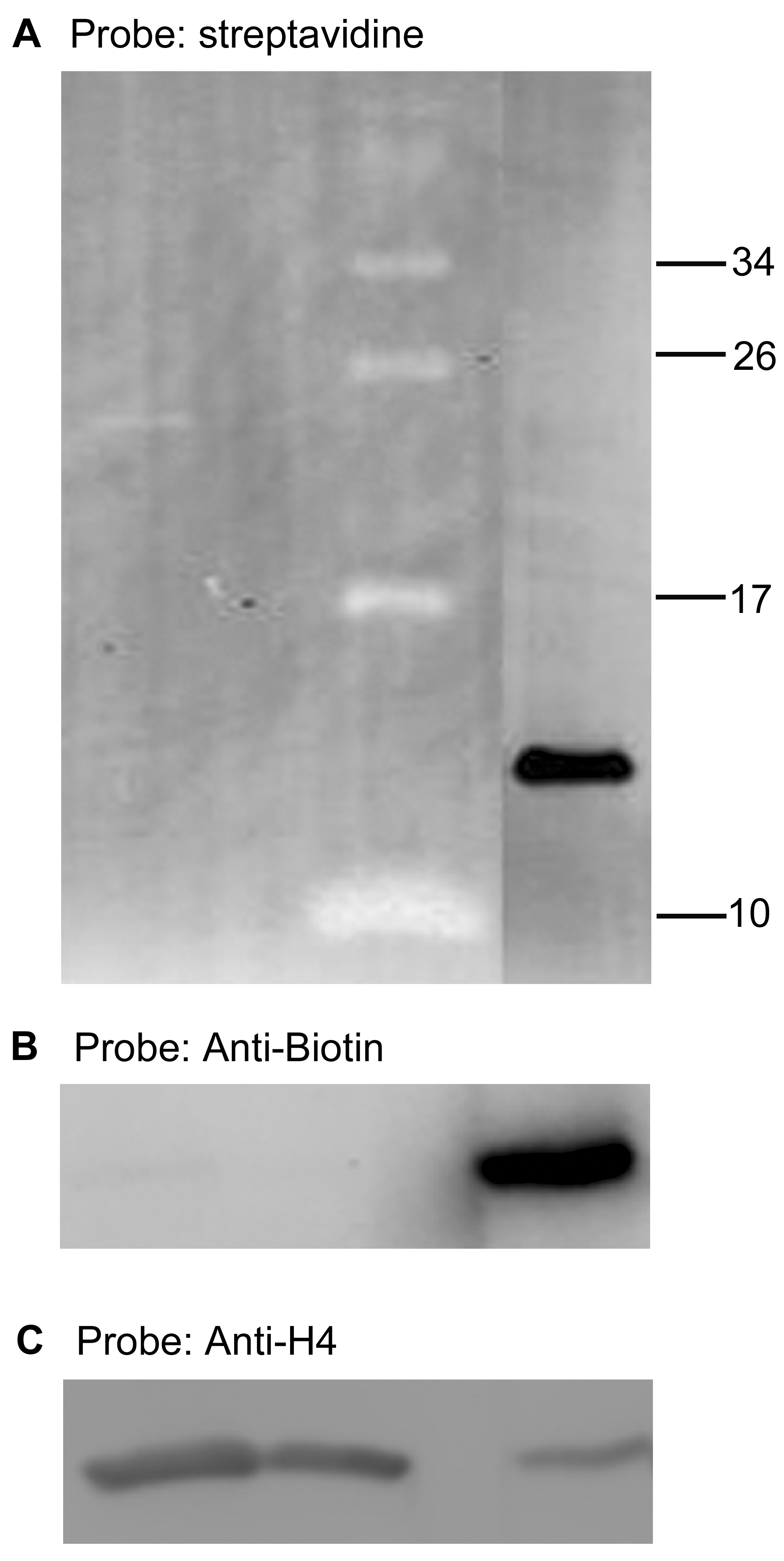

Supplement: Figure S1 — Testing of different samples of K12C H4 histone for biotinylation state. K12C-H4 was purified after overexpression in E.coli (lane1), depleted for fraction possibly biotinylated in vivo at lysines (lane 2) and biotinylated in vitro at cysteine 12 with Maleimide-PEG2-Biotin (lane 3). The level of chemical biotinylation was assessed by Western blotting with streptavidin conjugates (panel a) and anti-biotin antibodies (panel b). Control western blot with anti-H4 antibodies (panel c) demonstrates that all three samples in lanes 1-3 are histone H4. M - marker. (TIF) [file pone.0016299.s001.tif]

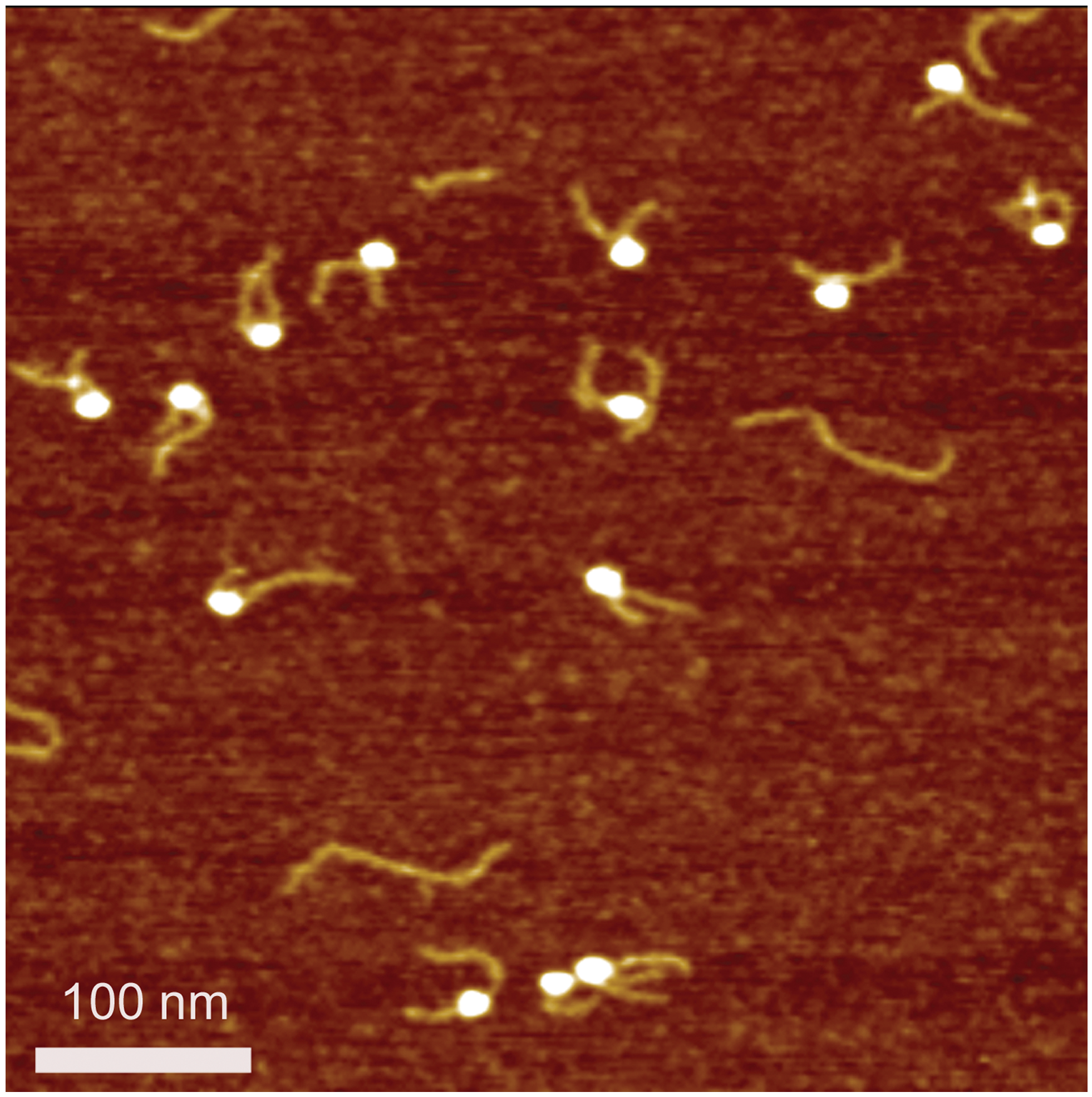

Supplement: Figure S2 — Representative AFM scan of nucleosome core particles reconstituted with K12C H4 mutant. Nucleosomes were made with K12C-H4 histone mutant. The sample was prepared and imaged as described for Figure 2. The image represents nucleosomes with different amount of DNA wrapped around the core particle. K12Cbio-H4 nucleosome conformation is similar to nucleosomes made with native histone H4. Scan size is 0.5 µm. (TIF) [file pone.0016299.s002.tif]

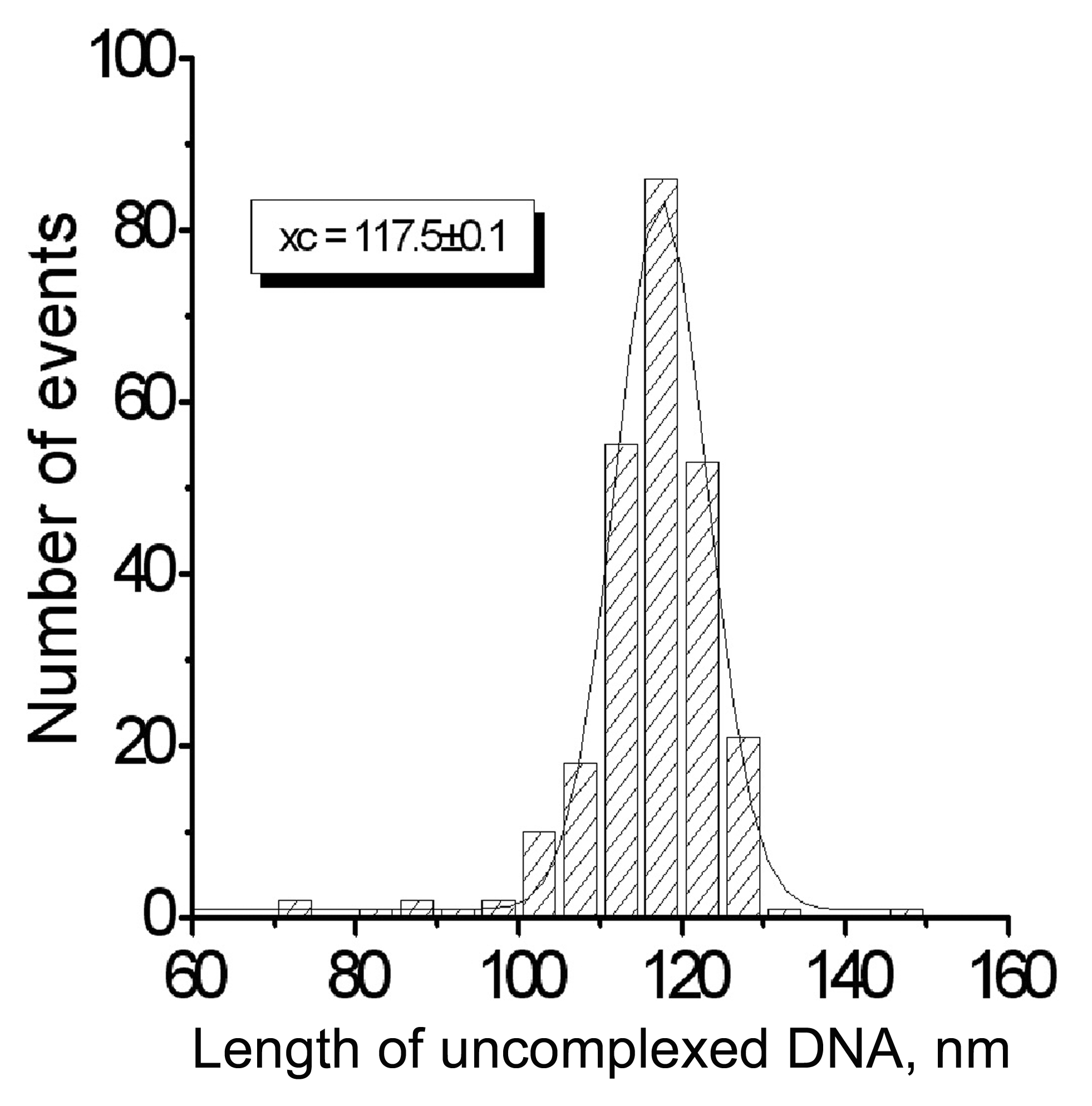

Supplement: Figure S3 — Length of uncomplexed DNA. Shown is distribution of length of uncomplexed DNA used for reconstitution of nucleosome core particles. The length of DNA was measured with FemtoScan software using parameter “curve”. The data were plotted as statistical histogram and fitted with Gaussian distribution. The most probable value of 117.5±0.1 nm was taken as length of full DNA molecule in subsequent calculations of length of DNA wrapped around nucleosome. (TIF) [file pone.0016299.s003.tif]

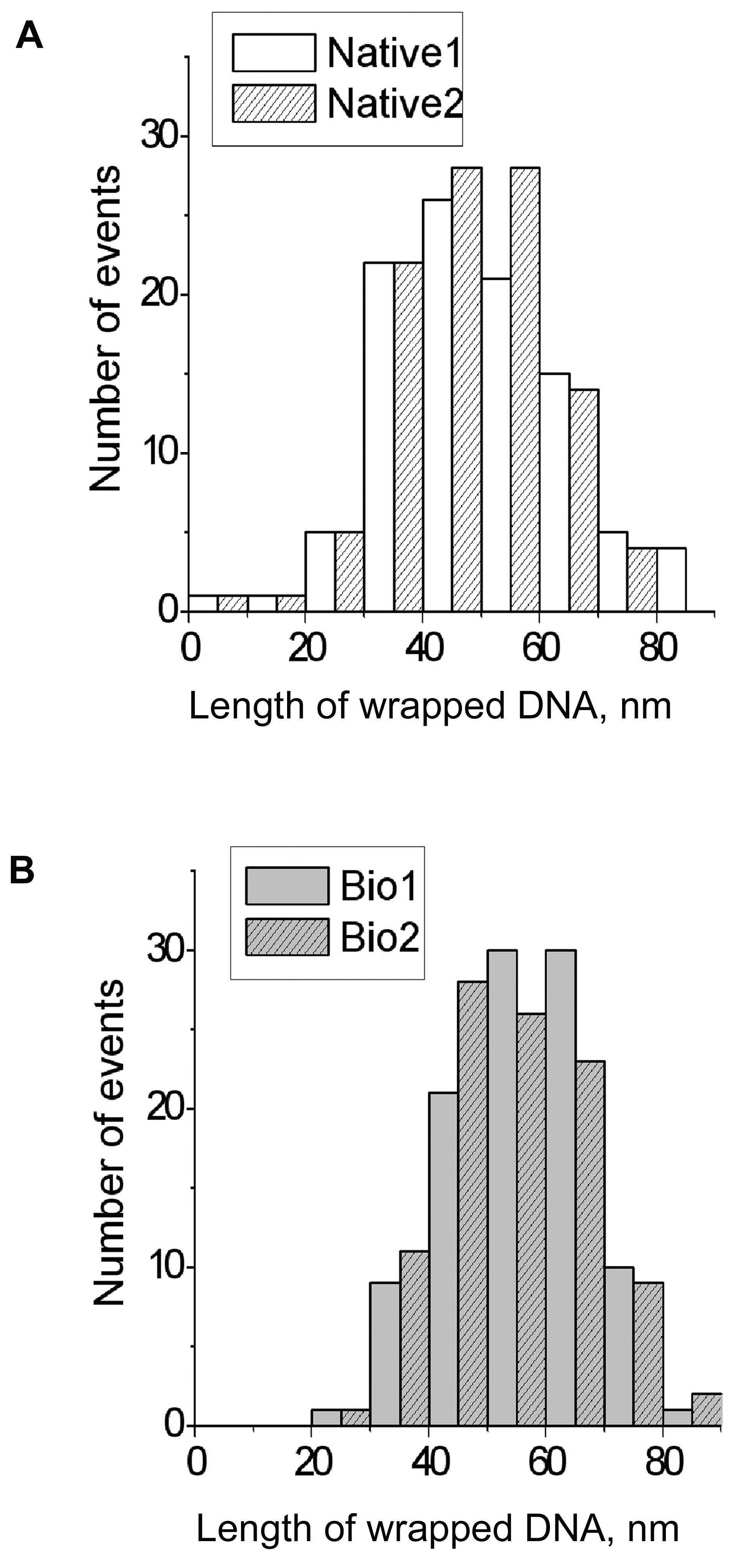

Supplement: Figure S4 — Comparison of wrapped DNA length from two independent samples of native and biotinylated H4 nucleosomes. Nucleosomes were reconstituted using native H4 histone (a) or biotinylated K12Cbio-H4 histone (b). Length of free DNA was measured using Femtoscan software. The length of wrapped DNA was calculated by subtracting the sum of both free DNA hands from total length of DNA. Data from two independent experiments are overlapped. It can be seen that in nucleosomes made with K12Cbio-H4 wDNA is shifted towards higher value compared to samples reconstituted using non-biotinylated native H4. Mean values for native NCP wDNA were 49.8±1.5 and 47.5±1.3 nm, respectively. Mean values for wDNA of biotinylated NCP (K12Cbio-H4) were 56.6±1.1 and 54.2±1.2 nm, respectively. (TIF) [file pone.0016299.s004.tif]
