# Supplementary material for: The Role of Histone H4 Biotinylation in the Structure of Nucleosomes
Source: PLoS One. 2011 Jan 27;6(1):e16299. doi: 10.1371/journal.pone.0016299 (PMC3029316; doi:10.1371/journal.pone.0016299)
Supplement: Materials and Methods S1 — Supplement to Materials and Methods. (DOC) [file pone.0016299.s005.doc]

**Supporting information.**

**Materials and methods**

1. **Histone octamer assembly and purification.**

Eighty µg of each histone H2A, H2B, H3 and H4 were individually concentrated 3-fold with Microcon filters and denatured in unfolding buffer containing 6 M guanidine HCl, 20 mM Tris-HCl and 5 mM DTT buffer for 2.5 h. Histones were mixed in equimolar ratios (typically 5-7 nmoles each) and concentration was adjusted to 1 mg/ml either by dilution with unfolding buffer or by centrifugation. The histone mixture was dialyzed at 4^o^C using Slide-A-Lyzer dialysis cassette with molecular weight cutoff 7,000 (Pierce) against three changes of 250 µl of refolding buffer containing 2 M NaCl, 10 mM Tris-HCl, pH 7.5, 1 mM Na-EDTA and 5 mM 2-ME.

1. **Estimating of the number of DNA turns**.

To assign specific number of turns the following issues were taken into account.

1. The expected length of wrapped DNA was calculated for specific measured rotation angle α using formula: 0.081(nm/0)*α, where 0.081 relates angle and length of wrapped DNA and comes from formula 1 turn= 360o = 29 nm. For molecules with expected 1 and 2 basic turns the total expected length was calculated with formulas [29 nm + 0.081(nm/o)*α] and [58 nm + 0.081(nm/o)*α], respectively.
2. Difference between measured length and each of two total expected lengths was calculated and rough assignment of 1 or 2 turns was made based on whichever difference between measured and calculated length was smaller. 3) Finally, the refined turn value was calculated with formula (360*n+ α)/360, where n is number of turns from rough assignment as explained above and equals to 1 or 2. Parameter of volume was also used as an auxiliary parameter when assigning number of turns.
